# The Doctor as a Business Man

**Published:** 1894-01

**Authors:** 


### THE DOCTOR AS A BUSINESS MAN.

The doctor, especially the country doctor, is, as a rule, a poor business man. In so being, he works an injustice to himself, his family and to his patient.

Either from imagined want of time for the business part of his work, fear of offending somebody, or a too low estimate of the value of his services, he neglects his accounts and takes, with thanks, what remuneration he gets, as if it were a lucky draft in the lottery of Fortune. Yet, he anticipates many a bill, lives up to what he "ought to collect," and in the end finds himself poor and in debt. His family has to keep up a shabby-genteel appearance and "live close" to keep within his means.

The patient suffers through the doctor's not making him pay, by feeling himself an unwelcome visitor, and perhaps finds the service rendered given in a grudging, half-hearted manner.

The time and money spent by a doctor in getting his education, his equipment for the actual practice, and the sacrifice of his time to the present or prospective demands of patients constitute his capital.

A good living interest should be drawn from this capital, and more if the work he does entitles him to it.

No good man will be offended at the presentation of a just bill, and it should be just, and no good doctor wants the practice of a good-for-nothing patient, unless in pure charity. And don't let yours be the only charity. If a patient pays railroads, hotels, hacks, druggists, living expenses, make him pay you.

Be business men, and dignify your profession by following business principles.

